# MRI-based Histopathological Imaging Features Predict Molecular Subtypes of Breast Cancer

**DOI:** 10.2174/0115734056411259251028093038

**Published:** 2025-11-28

**Authors:** Huibin Zhang, Yinfeng Qian

**Affiliations:** 1 Department of Radiology, The First Affiliated Hospital of Anhui Medical University, Hefei, 230022, China; 2 Department of Radiologic Imaging, Taihe County People's Hospital, Taihe, Anhui Province, 236600, China

**Keywords:** Breast cancer, MRI, Molecular classification, Magnetic resonance imaging, Immunohistochemistry, Tumors

## Abstract

**Introduction:**

This study aimed to investigate the correlation between magnetic resonance imaging (MRI) characteristics and molecular subtypes of breast carcinoma.

**Methods:**

A retrospective analysis was carried out on 194 breast cancer patients who underwent preoperative MRI. Pathological confirmation and molecular subtyping were performed on postoperative specimens. Preoperative MRI features of the lesions were evaluated. Univariate and multivariate logistic regression analyses were employed to identify MRI features associated with each molecular subtype.

**Results:**

A total of 194 breast cancer patients who underwent preoperative MRI and surgical treatment were included, with a mean age of 52.31 ± 12.08 years. Invasive ductal carcinoma was the predominant diagnosis (94.84%), and the expression rates of ER, PR, and HER2 were 58.76%, 55.67%, and 35.05%, respectively. The Ki-67 index was >20% in 70.62% of patients. Luminal B (HER2−) was the most common molecular subtype (33.51%). Significant differences were observed in lesion morphology, T2-weighted signal intensity, enhancement pattern, and type across the five molecular subtypes, though delayed-phase enhancement kinetics showed no significant variation. Logistic regression indicated that low T2WI signal and restricted diffusion were associated with Luminal A, while mass-like morphology and delayed-phase washout were predictors of Luminal B. Non-Mass Enhancement (NME) and rapid early enhancement were linked to HER2-enriched tumors, and unifocal, high T2WI signal, delayed-phase washout, and irregular margins were characteristic of triple-negative breast cancer.

**Conclusion:**

Distinct MRI features were found to be associated with specific molecular subtypes of breast cancer, providing valuable insights for subtype-specific diagnosis and therapeutic strategy formulation.

## 1 INTRODUCTION

Breast cancer is the most prevalent malignancy among women, with the highest incidence rate and the fourth leading cause of cancer-related mortality globally. Epidemiological studies reported that in 2022, approximately 2.3 million new cases were diagnosed worldwide [1]. Breast cancer is a multifactorial and biologically complex disease, driven by the interplay of genetic predisposition, environmental exposures, and lifestyle influences [2]. It exhibits marked heterogeneity, comprising distinct molecular subtypes characterized by differential expression of estrogen receptor (ER), progesterone receptor (PR), and human epidermal growth factor receptor 2 (HER2) [3]. Ki-67 is a well-established proliferation marker frequently utilized in the clinical classification of breast cancer subtypes [4]. Breast cancer is classified into five distinct intrinsic subtypes: Luminal A, Luminal B, HER2-enriched, basal, and normal-like [5, 6]. These subtypes exhibit variations in prognosis, clinical characteristics, and treatment responses [7, 8]. Recent research has revealed substantial inter-patient variability even within the same molecular subtype of breast cancer [9]. Intratumoral heterogeneity has emerged as a critical contributor to disease progression, therapeutic resistance, and tumor recurrence. Findings from a cohort study indicated that radiotherapy markedly reduced the risk of tumor metastasis and was associated with improved prognosis in patients with the tubulointerstitial type A phenotype [10, 11]. Therefore, determining the molecular subtype of breast cancer is important for subsequent targeted therapy.

Radiomics is an emerging non-invasive technique that enables the extraction of high-throughput quantitative features from medical images across various modalities, facilitating comprehensive characterization of tumor phenotypes and spatial heterogeneity [12, 13]. Early detection and intervention remain critical for enhancing patient survival outcomes. Currently, mammography is the most widely utilized modality for breast cancer screening; however, its limited sensitivity and specificity hinder the accurate differentiation between malignant and benign lesions, posing challenges for early diagnosis due to suboptimal image quality [14]. In contrast, Magnetic Resonance Imaging (MRI) offers high-resolution, three-dimensional anatomical visualization and is the most sensitive and specific imaging modality for lesion detection and diagnostic assessment [15]. MRI has been extensively applied in the evaluation of breast cancer. A retrospective multicenter study demonstrated that radiomic features derived from MRI hold significant promise for predicting axillary lymph node (ALN) metastasis in early-stage invasive breast cancer [16]. Furthermore, the integration of artificial intelligence (AI) with MRI can substantially reduce unnecessary diagnostic procedures, while the combination of MRI with other imaging modalities notably enhances diagnostic sensitivity [17, 18]. Emerging evidence suggests that tumor radiomic signatures may correlate with distinct biological characteristics [19]. In addition, it suggests that algorithmic optimization may enhance model performance, potentially serving as an alternative approach for molecular subtyping prediction [20, 21]. However, limited research has focused on utilizing MRI to differentiate molecular subtypes of breast cancer, underscoring the need for further investigation into the imaging phenotypes associated with various breast cancer subtypes.

This study aims to investigate the correlation between molecular subtypes of breast cancer and MRI-derived imaging features by constructing and analyzing MRI-based imaging models derived from surgical specimens.

## 2 METHODS

### 2.1 Study Population

Patients with histopathologically confirmed breast cancer diagnosed between February 2021 and February 2024 were retrospectively enrolled for analysis. All patients underwent breast MRI, including dynamic contrast-enhanced (DCE) sequences, within 3 weeks of surgery. Exclusion criteria were as follows: (1) prior history of radiotherapy, chemotherapy, or recurrent breast cancer; (2) incomplete or non-diagnostic MRI data; and (3) inadequate tumor tissue for molecular subtype classification. The study was ethically reviewed by the People's Hospital of Taihe County. Human subjects were treated in accordance with the Declaration of Helsinki.

### 2.2 Identification of Molecular Subtypes of Breast Cancer

Immunohistochemistry (IHC) was employed to evaluate the expression of ER, PR, HER2, and the Ki-67 proliferation index. ER and PR positivity were defined as nuclear staining in ≥1% of tumor cells in high-power fields (×400 magnification). HER2 positivity was determined as either a score of 3+ on IHC or a score of 2+ on IHC with confirmed HER2 gene amplification by fluorescence *in situ* hybridization (FISH). Breast cancers were classified into five molecular subtypes as follows: (1) Luminal A: ER-positive and/or PR-positive, HER2-negative, and Ki-67 ≤ 20%; (2) Luminal B (HER2-negative): ER-positive and/or PR-positive, HER2-negative, and Ki-67 > 20%; (3) Luminal B (HER2-positive): ER-positive and/or PR-positive, HER2-positive, regardless of Ki-67 index; (4) HER2-enriched: ER-negative, PR-negative, HER2-positive, regardless of Ki-67 index; (5) Triple-negative: ER-negative, PR-negative, and HER2-negative, regardless of Ki-67 expression. HER2-positive breast cancers were further subclassified into HER2+/HR- and HER2+/HR+ based on hormone receptor status [22].

### 2.3 MRI Examination and Feature Extraction

Breast MRI examinations were performed using the United Imaging UMR770 3.0T MRI system. The MRI scanning sequences included: (1) T1-weighted imaging (T1WI) using fast spin-echo sequences; (2) T2-weighted imaging (T2WI) using fast spin-echo sequences (TR = 4948 ms, TE = 87.2 ms; slice thickness = 4 mm); (3) T2 short-tau inversion recovery (STIR) using fast spin-echo sequences (TR = 4948 ms, TE = 87.2 ms; slice thickness = 4 mm); (4) T1-weighted quick 3D dynamic contrast-enhanced sequence (TR = 3.9 ms, TE = 1.45 ms; slice thickness = 2 mm), with imaging performed before and after contrast injection at six time points (contrast agent dose: 0.2 mL/kg, injection rate: 3 mL/s); (5) Diffusion-weighted imaging (DWI) using three b-values (0, 50, 800 s/mm^2^) (TR = 4072 ms, TE = 69.4 ms; slice thickness = 4 mm).

Two radiologists with 10 and 5 years of experience, respectively, independently evaluated the MRI features of breast lesions according to the 2013 edition of the Breast Imaging Reporting and Data System (BI-RADS), with blinding to the pathological outcomes [23]. The final assessment was based on consensus between the two radiologists. MRI features evaluated included: lesion type, mass shape and margins, distribution of non-mass enhancement (NME), number of lesions, T2-weighted signal intensity, presence of diffusion restriction, and enhancement patterns. A region of interest (ROI) was manually delineated in the area of maximal lesion enhancement. From this ROI, the early enhancement rate of the dynamic enhanced contrast (DEC) and the time–intensity curve (TIC) were extracted to assess the kinetic enhancement pattern. Early enhancement was categorized as slow (<50%), intermediate (50%–100%), or rapid (>100%). The delayed phase TIC was classified into three types: (1) Persistent: signal intensity increases >10%; (2) Plateau: signal intensity change ≤10%; (3) Washout: signal intensity decreases >10%. All immunohistochemical assays were performed using validated antibodies (ER: SP1 clone, Ventana; PR: 1E2 clone, Roche; HER2: 4B5 clone, Ventana; Ki-67: 30-9 clone, Ventana) on automated staining platforms with appropriate positive and negative controls. HER2 testing was performed according to the 2018 ASCO/CAP guidelines, utilizing the Ventana INFORM HER2 Dual ISH DNA Probe for equivocal cases (IHC 2+), with amplification defined as a HER2/CEP17 ratio of ≥2.0.

### 2.4 Statistical Analyses

All statistical analyses were performed using R software. Statistical information is presented as Mean ± SD, while categorical variables are expressed as percentages. Differences in categorical variables were assessed using the Chi-square test or Fisher’s exact test, as appropriate. Logistic regression models were employed to investigate the associations between radiomics features and molecular subtypes. A *p*-value of less than 0.05 was considered indicative of statistical significance.

## 3 RESULTS

### 3.1 Patient Baseline Information

A total of 194 patients who underwent preoperative breast MRI and surgical treatment for breast cancer were included in this study, with a mean age of 52.31 ± 12.08 years. All participants were female. Details are presented in Table **1**. The majority of patients were diagnosed with invasive ductal carcinoma (94.84%). Positive expression rates for ER, PR, and HER2 were 58.76%, 55.67%, and 35.05%, respectively. The Ki-67 proliferation index was greater than 20% in 70.62% of patients. In terms of molecular subtypes, the most common was Luminal B (HER2−) (33.51%), while the least common was HER2+/HR+ (11.34%).

### 3.2 Correlation of MRI Imaging Features with Different Molecular Subtypes

Significant differences were observed among the five molecular subtypes with respect to lesion morphology, multiplicity, T2-weighted signal intensity, enhancement pattern, and enhancement type, while no significant variation was found in the delayed-phase enhancement kinetics (Table **2**). Additionally, within the HER2-positive subgroup, lesion type and margin showed significant differences; mass-like lesions were more prevalent in both HR− and HR+ groups, and irregular margins were more frequently observed, particularly in HR+ tumors (Table **3**).

**Fig. (1):**
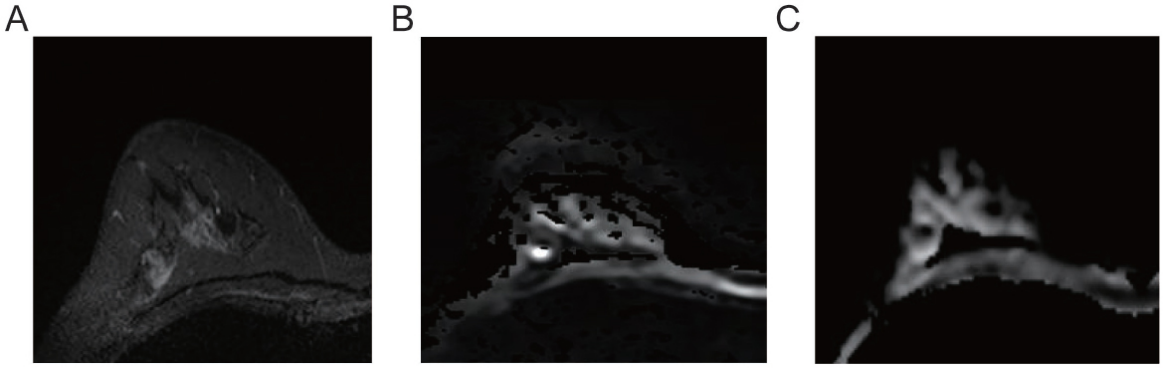
Right breast ductal carcinoma *in situ* (Luminal A subtype). The lesion appears as isointense on T2-weighted imaging **(A)**. Diffusion-weighted imaging (DWI, b=800) shows high signal intensity **(B)**, while ADC imaging demonstrates slightly increased signal intensity **(C)**.

**Fig. (2):**
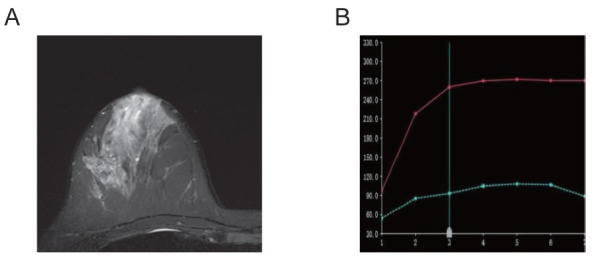
Right breast ductal carcinoma *in situ* (Luminal B subtype, HER2-negative). The lesion presents as a mass with heterogeneous, high signal intensity on T2-weighted imaging **(A)**. The TIC demonstrates slow-to-moderate early enhancement **(B)**.

**Fig. (3):**
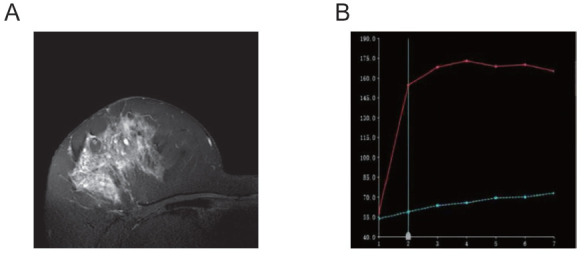
Right breast ductal carcinoma *in situ* (HER2-enriched subtype). The lesion is of the non-mass enhancement (NME) type, with heterogeneous high signal intensity on T2-weighted imaging **(A)**. The TIC shows rapid early enhancement **(B)**.

**Fig. (4):**
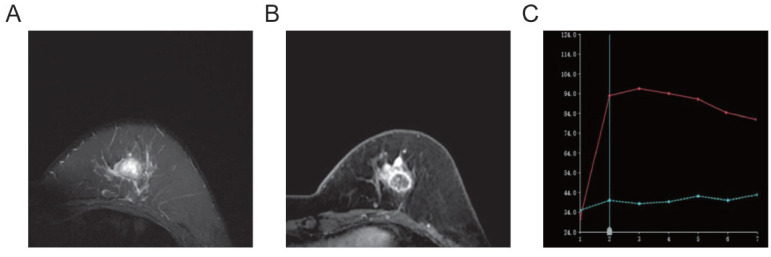
Left breast ductal carcinoma *in situ* (triple-negative subtype). The lesion shows high signal intensity at the center and iso- to low signal intensity at the margins on T2-weighted imaging **(A)**. Enhancement of the lesion reveals a ring-like pattern **(B)**. The TIC demonstrates an outflow-type enhancement **(C)**.

Logistic regression analysis revealed that lesion type, edge enhancement, and the Luminal A subtype exhibited a negative association; however, these findings did not reach statistical significance after adjustment for covariates. In contrast, low or iso-intense T2-weighted imaging (T2WI) signal and diffusion restriction (OR=3.574, 95% CI=1.621-7.883, *p*=0.024), indicated by low apparent diffusion coefficient (ADC) values (OR=3.721, 95% CI=1.655-8.372, *p*=0.013), were significantly associated with an increased likelihood of the Luminal A subtype (Fig. **1A-C**). Moreover, mass-like morphology and delayed-phase washout emerged as potential independent predictors of the Luminal B (HER2−) subtype (Fig. **2A-B**). For the HER2-enriched subtype, non-mass enhancement (NME) and early rapid contrast uptake were strongly correlated (OR>1) (Fig. **3A-B**). In triple-negative breast cancer, key imaging features included unifocal presentation, high T2WI signal intensity, delayed-phase washout, and irregular lesion margins (OR>1) (Fig. **4A-C**, Table **4**). Additionally, irregular mass-like lesions were significantly more frequent in the HER2+/HR+ subtype compared to the HER2+/HR− subtype (OR=6.146, 95% CI=1.215-31.07, *p*=0.028) (Table **5**).

## 4 DISCUSSION

Breast cancer remains the most prevalent malignancy and the leading cause of cancer-related mortality among women globally [24, 25]. It is a biologically heterogeneous disease, characterized by distinct molecular subtypes that differ in risk factors, clinical presentation, prognosis, and therapeutic strategies [26, 27]. Identifying reliable imaging and molecular markers for these subtypes is essential for optimizing early detection, individualized treatment planning, and improving overall patient outcomes. Imaging histology is an emerging noninvasive technique for characterizing oncological features [28]. Among these modalities, MRI has been extensively applied in oncology. Recent research has demonstrated the utility of MRI-based radiomic models for pre-treatment risk stratification of endometrial cancer [29]. In the context of breast cancer, prospective studies have shown that MRI-derived imaging biomarkers can effectively predict heterogeneous HER2 expression [30]. These studies underscore the pivotal role of MRI in the diagnosis, characterization, and management of tumors.

In this study, we observed that MRI features were associated with various molecular subtypes of breast cancer. The Luminal A subtype typically exhibited relatively uniform or low T2WI signals, with non-diffusion-limited characteristics, whereas the Luminal B (HER2-) subtype displayed a more mass-like pattern. Previous research has indicated that Luminal A lesions are highly differentiated, predominantly solid, and demonstrate reduced neovascularization and intra-lesional fibrosis. Additionally, patients with Luminal A breast cancer tend to have a better prognosis compared to other subtypes [31]. Furthermore, it has been shown that low T2WI values are particularly useful for distinguishing Luminal A lesions [32]. Another study demonstrated that ADC values exhibit overlap across different molecular subtypes of breast cancer. These values can be used to develop various predictive models for assessing the molecular subtypes of breast cancer [33]. In contrast, Luminal B (HER2-) lesions are characterized by a high Ki-67 proliferation index and increased cell density, with a positive correlation between mass-like growth and elevated Ki-67 levels [23]. The association between slow-to-moderate early intensification and Luminal B (HER2-) was statistically significant, consistent with prior research. This suggests that HER2 expression may influence DCE hemodynamic patterns [34]. HER2, an oncogene that promotes tumor cell proliferation, is overexpressed in approximately 15% of breast cancers and serves as a critical therapeutic target [35, 36]. Elevated HER2 levels are linked to high vascular endothelial growth factor (VEGF) expression, which increases vascular permeability. This mechanism could help explain the observed early intensification pattern [37]. Furthermore, although the Luminal B (HER2+) subtype did not exhibit significant MRI characteristics, the HER2+/HR+ subtype demonstrated more mass-like and irregularly bordered lesions compared to the HER2-enriched (HER2+/HR-) type. These findings are in line with previous studies, which have reported that HR-positive tumors tend to show enhanced interstitial tumor responses, increased fibrosis, and peripheral burr formations [38]. Additionally, our findings indicated that HER2-enriched lesions were more frequently associated with non-mass enhancement (NME) patterns and exhibited rapid early enhancement. High HER2 expression was correlated with a lower histological grade and facilitated tumor metastasis, potentially promoting neovascularization within the lesions [39, 40]. This neovascularization could enhance early contrast uptake, contributing to the observed rapid enhancement pattern. We also found no significant differences in delayed-phase enhancement kinetics among breast cancer molecular subtypes. This may be because the delayed phase primarily reflects vascular permeability and interstitial pressure equilibrium, while vascular permeability differences between subtypes are more pronounced in the early phase. A retrospective study similarly demonstrated no significant differences in delayed washout enhancement patterns among different subtypes [41]. Our analysis revealed no significant association between lesion morphology/marginal enhancement and Luminal A subtype after covariate adjustment. This finding may be attributed to the favorable prognosis of Luminal A tumors, which typically exhibit well-circumscribed, nodular growth patterns [42]. These observations are supported by prior research that similarly reports non-significant differences in enhancement margins across molecular subtypes [21].

Triple-negative breast cancer (TNBC) is a heterogeneous and highly aggressive tumor subtype, characterized by poor prognosis and a high propensity for metastasis and recurrence [43]. Compared to other subtypes, TNBC often presents with relatively benign imaging features [44, 45]. It frequently develops unifocal infiltrative lesions with high T2WI signal intensity, suggesting intratumoral necrosis. This observation is consistent with findings from several studies [34, 46]. This study also identified that edge enhancement and outflow-type delayed enhancement were associated with an increased risk of TNBC. Retrospective analysis revealed that angiogenesis was more pronounced at the margins of TNBC lesions, and there was a noticeable difference in fibrosis between the margins and the lesion center. This disparity may significantly contribute to the observed margin enhancement [47]. Moreover, given the highly invasive nature of TNBC, although angiogenesis within the lesion is active, the vascular structures are irregular and disorganized, leading to ineffective blood perfusion. As a result, this tumor typically demonstrates an outflow-type enhancement pattern. The correlation between this hemodynamic feature, the degree of malignancy in TNBC, and its prognostic implications has been highlighted [48]. All of the aforementioned findings underscore the strong correlation between MRI imaging features and the various molecular subtypes of breast cancer. This relationship extends beyond merely subtype identification; it also holds potential for the development of novel diagnostic tools and therapeutic targets, which is of significant importance.

This study employed radiomics analysis to characterize molecular subtype differences and identify key imaging features, providing a theoretical foundation for screening early breast cancer. Future research may focus on AI-driven automated feature extraction *via* segmentation techniques to reduce clinician workload [49, 50].

### 4.1 Study Limitation

This study has several limitations. First, the retrospective design and single-center data source may introduce inherent selection bias. Second, the limited number of patients presenting with non-mass enhancement (NME) reduces the statistical power for subgroup analyses. In addition, the three-week interval between the MRI examination and surgery may affect the results due to changes in tumor morphology, thereby reducing their accuracy. Third, the evaluation of DCE-MRI morphological characteristics and ADC values may be subject to interobserver variability, potentially influencing the robustness of the findings. To address these limitations, future studies should incorporate larger-scale, multicenter prospective cohorts with adequately powered sample sizes (≥50 cases per molecular subtype) and rigorous matching for potential confounders, such as age. Molecular subtyping should employ high-throughput sequencing and computer-aided diagnostic systems to minimize interobserver variability and subjective bias.

## CONCLUSION

In summary, our findings reveal a significant association between MRI-derived imaging features, such as T2-weighted signal intensity and lesion morphology, and breast cancer molecular subtypes. These imaging biomarkers may serve as valuable adjuncts to immunohistochemical profiling, offering potential utility in guiding individualized treatment strategies. Nonetheless, further validation in large-scale, prospective studies is essential before clinical translation.

## Figures and Tables

**Table 1:** Baseline characteristics of the study population.

Characteristic	Number	Percentage (%)
**Histopathological Type**	-	-
Invasive Ductal Carcinoma	184	94.84
Ductal Carcinoma *In Situ*	8	4.13
Invasive Lobular Carcinoma	1	0.52
Mucinous Carcinoma	1	0.52
**Hormone Receptor Status**	-	-
ER Positive (ER+)	114	58.76
PR Positive (PR+)	108	55.67
HER2 Positive (HER2+)	68	35.05
**Ki-67 Proliferation Index**	-	-
≤ 20%	57	29.38
> 20%	137	70.62
**Molecular Subtypes**	-	-
Luminal A	36	18.56
Luminal B (HER2−)	65	33.51
Luminal B (HER2+)	30	15.46
HER2-Enriched (HER2+/HR−)	38	19.59
Triple-Negative	25	12.89
HER2+/HR+	22	11.34

**Table 2:** Comparison of MRI features across five breast cancer molecular subtypes.

**MRI Feature**	**Luminal A (n=36)**	**Luminal B (HER2−) (n=65)**	**Luminal B (HER2+) (n=30)**	**HER2-Enriched (n=38)**	**Triple-Negative (n=25)**	***p***
**Lesion Morphology**	-	-	-	-	-	1.76×10^-4^
Mass-like lesion	23 (63.9%)	61 (93.8%)	27 (90.0%)	25 (65.8%)	22 (88.0%)	-
NME	13 (36.1%)	4 (6.2%)	3 (10.0%)	13 (34.2%)	3 (12.0%)	-
**Lesion Multiplicity**	-	-	-	-	-	1.18×10^-2^
Unifocal	15 (41.7%)	29 (44.6%)	10 (33.3%)	11 (28.9%)	18 (72.0%)	-
Multifocal	21 (58.3%)	36 (55.4%)	20 (66.7%)	27 (71.1%)	7 (28.0%)	-
**T2WI Signal Intensity**	-	-	-	-	-	7.14×10^-3^
High signal intensity	11 (30.6%)	30 (46.2%)	12 (40.0%)	21 (55.3%)	19 (76.0%)	-
Iso-/Low signal intensity	25 (69.4%)	35 (53.8%)	18 (60.0%)	17 (44.7%)	6 (24.0%)	-
**Diffusion Restriction (DWI/ADC)**	-	-	-	-	-	2.31×10^-2^
Restricted diffusion	30 (83.3%)	61 (93.9%)	27 (90.0%)	38 (100.0%)	25 (100.0%)	-
No restriction	6 (16.7%)	4 (6.1%)	3 (10.0%)	0 (0.0%)	0 (0.0%)	-
**Early Enhancement Pattern**	-	-	-	-	-	1.73×10^-3^
Rapid	17 (47.2%)	25 (38.5%)	18 (60.0%)	29 (76.3%)	17 (68.0%)	-
Medium or slow	19 (52.8%)	40 (61.5%)	12 (40.0%)	9 (23.7%)	8 (32.0%)	-
**Delayed Enhancement Curve**	-	-	-	-	-	1.92×10^-1^
Washout	13 (36.1%)	26 (40.0%)	11 (36.7%)	18 (47.4%)	16 (64.0%)	-
Plateau/persistent	23 (63.9%)	39 (60.0%)	19 (63.3%)	20 (52.6%)	9 (36.0%)	-
**Enhancement Type**	-	-	-	-	-	4.36×10^-3^
Rim enhancement	6 (16.7%)	19 (29.2%)	10 (33.3%)	13 (34.2%)	16 (64.0%)	-
Non-rim enhancement	30 (83.3%)	46 (70.8%)	20 (66.7%)	25 (65.8%)	9 (36.0%)	-

**Table 3:** Comparison of MRI features between different subtypes of HER2+.

**MRI Characteristic**	**HER2+/HR− (n = 38)**	**HER2+/HR+ (n = 22)**	***p***
**Lesion Morphology**	-	-	3.29×10^-3^
Mass-like	25 (65.8%)	21 (95.5%)	-
NME	13 (34.2%)	1 (4.5%)	-
**Lesion Margin (Mass)**	-	-	8.01×10^-2^
Round or Oval	9 (36.0%)	2 (9.5%)	-
Irregular	16 (64.0%)	19 (90.5%)	-

**Table 4:** Association between MRI features and breast vancer molecular subtypes.

**Subtype**	**MRI Feature**	**OR (95% CI) Univariate**	***p***	**OR (95% CI) Multivariate**	***p***
Luminal A	Lesion type	0.301 (0.134–0.678)	0	0.480 (0.209–1.102)	0.07
	Iso-/hypointense T2WI signal	2.452 (1.130–5.210)	0.02	3.574 (1.621–7.883)	0.02
	DWI restriction (low ADC)	4.314 (1.354–13.744)	0.01	3.721 (1.655–8.372)	0.01
	Margin enhancement	0.345 (0.135–0.878)	0.03	0.975 (0.587–1.617)	0.92
Luminal B (HER2−)	Lesion type	5.031 (1.695–14.928)	0	7.253 (1.672–31.472)	0.01
	Delayed-phase washout	2.791 (1.509–5.164)	0	4.172 (1.105–15.747)	0.03
HER2-enriched	NME	3.007 (1.347–6.713)	0.01	10.726 (2.348–48.681)	0
	Rapid initial enhancement	3.222 (1.432–7.251)	0.01	6.147 (2.074–18.241)	0
	Unifocal	4.114 (1.629–10.390)	0	5.826 (1.325–25.600)	0.02
Triple-negative	High T2WI signal	4.065 (1.546–10.690)	0	4.275 (1.077–16.947)	0.04
	Delayed washout	2.706 (1.121–6.479)	0.03	1.704 (1.035–2.803)	0.04
	Irregular margin	4.481 (1.854–10.831)	0	3.247 (1.020–10.339)	0.05
HER2+/HR- subtypes were used as a reference.	-	-	-	-	-

**Table 5:** Association between MRI features and HER2+ breast cancer molecular subtype.

**MRI Feature**	**OR (95% CI) Univariate**	***p***	**OR (95% CI) Multivariate**	***p***
Lesion type	10.92 (1.317–90.52)	0.03	7.352 (1.312–41.2)	0.02
Irregular margin	5.344 (1.006–28.395)	0.05	6.146 (1.215–31.07)	0.03
HER2+/HR- subtypes were used as a reference.	-	-	-	-

## Data Availability

All data generated or analyzed during this study are included in this published article.

## LIST OF ABBREVIATIONS

MRI: Magnetic resonance imaging
ER: Estrogen receptor
PR: Progesterone receptor
ALN: Axillary lymph node
AI: Artificial intelligence
DCE: Dynamic contrast-enhanced
IHC: Immunohistochemistry
FISH: Fluorescence *in situ* hybridization

## STANDARDS OF REPORTING

STROBE guidelines were followed.
